# Supplementary material for: Green Tea Catechins Modulate Skeletal Development with Effects Dependent on Dose, Time, and Structure in a down Syndrome Mouse Model
Source: Nutrients. 2022 Oct 7;14(19):4167. doi: 10.3390/nu14194167 (PMC9572077; doi:10.3390/nu14194167)
Supplement: Supplementary file 1 [file nutrients-14-04167-s001.zip › nutrients-1872891-supplementary.pdf]

SUPPLEMENTARY DATA

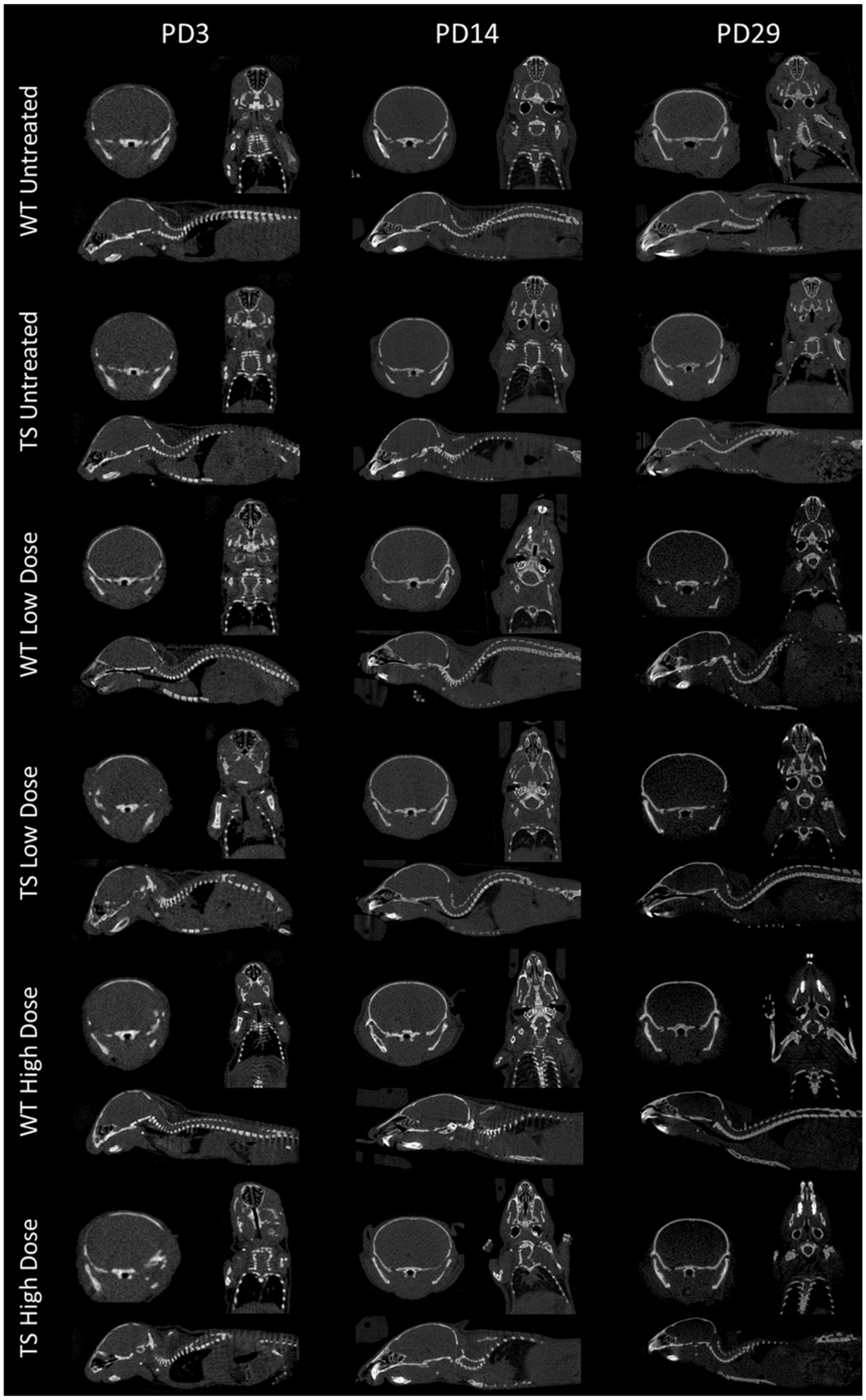

Supplementary Figure S1: Raw axial, coronal, and sagittal 2D projections from µCT scans throughout development. Images are shown for a wild-type untreated mouse, Ts65Dn untreated mouse, wild-type mouse treated with the low dose, Ts65Dn mouse treated with the low dose, wild-type mouse treated with the high dose and Ts65Dn mouse treated with the high dose. For each experimental group, the same mouse is shown at postnatal day (PD) 3 (left), PD14 (middle) and PD29 (right).

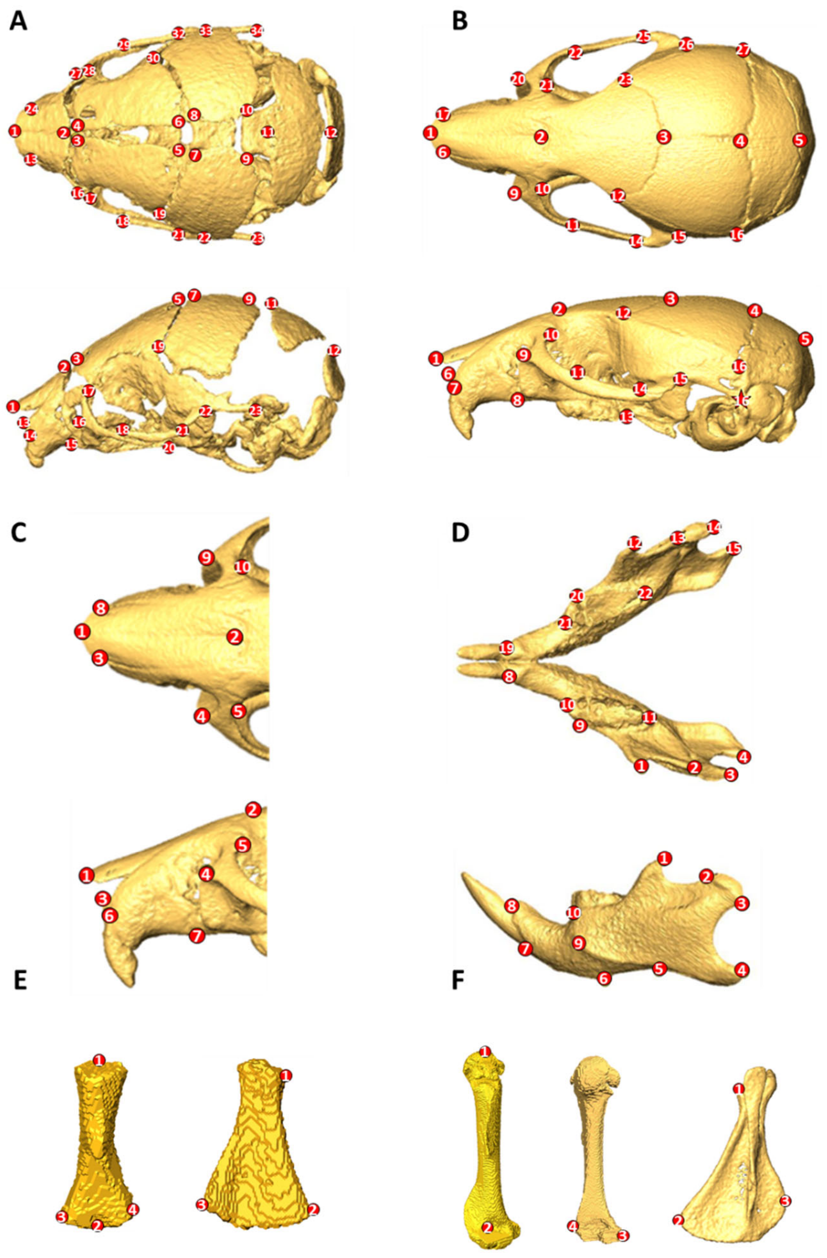

Supplementary Figure S2. Set of anatomical landmarks used to characterize the shape of the skull, mandible, scapula, and humerus. (A) Set of 34 landmarks characterizing skull shape at PD3 from a 3D reconstruction of a µCT scan. **(B)** Set of 27 landmarks characterizing skull shape at PD14 and PD29 from a 3D reconstruction of a µCT scan. **(C)** Set of 12 landmarks characterizing facial shape from a 3D reconstruction of a µCT scan. **(D)** Set of 22 landmarks characterizing mandible shape from a 3D reconstruction of a µCT scan. **(E)** Set of 4 landmarks used to measure length and width of the scapula (left) and humerus (right) at PD3. **(F)** Set of landmarks used to measure length and width of the scapula (left, middle) and humerus (right) at PD14 and PD29. See Supplementary Tables S3 to S7 for precise anatomical definitions.

Supplementary Table S1. Sample size for each analysis, experiment, and developmental stage. Differences in sample size between stages are due to technical reasons such as micro-CT or MR scanner not operating on the scanning day, movement scanning artifacts, or mouse death during the experiment.

	PD3				PD14				PD29			
	WT Untreated	TS Untreated	WT Treated	TS Treated	WT Untreated	TS Untreated	WT Treated	TS Treated	WT Untreated	TS Untreated	WT Treated	TS Treated
Face shape exp1	12	6	14	9	17	8	13	7	14	7	7	8
Skull shape exp1	12	6	15	9	17	8	13	7	14	7	7	8
Mandible shape exp1	12	6	14	9	17	7	13	7	14	6	12	8
Humerus length exp1	15	8	14	9	19	8	11	7	18	6	12	8
Humerus width exp1	15	8	13	9	19	8	11	7	18	6	12	8
Scapula length exp1	15	8	13	9	19	8	11	7	17	6	12	8
Scapula width exp1	15	8	13	9	19	8	11	7	17	6	12	8
BMD humerus exp1	18	9	12	9	18	8	6	6	15	4	12	8
BMD premaxilla exp1	19	8	13	9	18	8	6	6	14	4	10	8
Face shape exp2	15	8	15	6	17	6	15	6	15	6	15	5
Skull shape exp2	15	8	15	6	17	7	15	6	15	6	15	5
Mandible shape exp2	15	8	13	6	22	9	15	6	19	7	13	5
Humerus length exp2	15	8	15	6	19	8	15	6	18	6	15	5
Humerus width exp2	15	8	15	6	19	8	15	6	18	6	15	5
Scapula length exp2	15	8	15	6	19	8	15	6	17	6	15	5
Scapula width exp2	15	8	15	6	19	8	15	6	17	6	15	5
BMD humerus exp2	18	9	15	5	19	8	15	5	15	4	15	5
BMD premaxilla exp2	19	8	14	6	18	8	15	6	14	4	15	5

Supplementary Table S2. µCT scanning parameters used at each stage.

Stage	Source energy (Kv)	Filter	Current (µA)	Exposure time (ms)	Averages	Step increment (˚)	Total angle (˚)	Time	Voxel size (µm^3)	Radiation dose
PD3	35	Al 0.5 mm	500	180	3	1	180º	3 min	51.7	25 mGy
PD14	35	Al 0.5 mm	500	80	3	1	180º	3 min	51.7	110 mGy
PD29	55	Al 1 mm	700	80	2	1	360º	3 min	51.7	249 mGy

Supplementary Table S3. Anatomical definition of facial landmarks.

Landmark number	Anatomical definition
1	Tip of the nasal bone
2	Intersection of nasal and frontal bones (only visible at PD14 and PD29)
3	Anterior-most point on intersection of premaxillae and nasal bones (left)
4	Anterior notch on frontal process lateral to infraorbital fissure (left)
5	Intersection of frontal process of maxilla with frontal and lacrimal bones (left)
6	Center of alveolar ridge over maxillary incisor (left)
7	Most inferior point on premaxilla-maxilla suture (left)
8	Anterior-most point on intersection of premaxillae and nasal bones (right)
9	Anterior notch on frontal process lateral to infraorbital fissure (right)
10	Intersection of frontal process of maxilla with frontal and lacrimal bones (right)
11	Center of alveolar ridge over maxillary incisor (right)
12	Most inferior point on premaxilla-maxilla suture (right)
13	Posterior-most point on the nasal bone at the midline (only visible at PD3)
14	Anterior-most point on the frontal bone at the midline (left) (only visible at PD3)
15	Anterior-most point on the frontal bone at the midline (right) (only visible at PD3)

Supplementary Table S4. Anatomical definition of skull landmarks. N/A indicates that the landmarks were not acquired at this stage.

Landmark number at PD3	Landmark number at PD14 and PD29	Anatomical definition
1	1	Tip of the nasal bone
N/A	2	Intersection of nasal and frontal bones
N/A	3	Intersection of frontal and parietal bones
N/A	4	Intersection of parietal and interparietal bones
N/A	5	Intersection of interparietal and occipital bones
13	6	Anterior-most point on intersection of premaxillae and nasal bones (left)
14	7	Center of alveolar ridge over maxillary incisor (left)
15	8	Most inferior point on premaxilla-maxilla suture (left)
16	9	Anterior notch on frontal process lateral to infraorbital fissure (left)
17	10	Intersection of frontal process of maxilla with frontal and lacrimal bones (left)
18	11	Intersection of zygomatic process of maxilla with zygoma (left)
19	12	Frontal-squamosal intersection at temporal crest (left)
20	13	Intersection of maxilla and sphenoid on inferior alveolar (left)
21	14	Intersection of zygoma with zygomatic process of temporal (left)
N/A	15	Intersection of squamosal body to zygomatic process of squamosal (left)
N/A	16	Intersection of parietal, temporal and occipital bones (left)
24	17	Anterior-most point on intersection of premaxillae and nasal bones (right)
25	18	Center of alveolar ridge over maxillary incisor (right)
26	19	Most inferior point on premaxilla-maxilla suture (right)

27	20	Anterior notch on frontal process lateral to infraorbital fissure (right)
28	21	Intersection of frontal process of maxilla with frontal and lacrimal bones (right)
29	22	Intersection of zygomatic process of maxilla with zygoma (right)
30	23	Frontal-squamosal intersection at temporal crest (right)
31	24	Intersection of maxilla and sphenoid on inferior alveolar (right)
32	25	Intersection of zygoma with zygomatic process of temporal (right)
N/A	26	Intersection of squamosal body to zygomatic process of squamosal (right)
N/A	27	Intersection of parietal, temporal and occipital bones (right)
2	N/A	Posterior-most point on the nasal bone at the midline
3	N/A	Anterior-most point on the frontal bone at the midline (left)
4	N/A	Anterior-most point on the frontal bone at the midline (right)
5	N/A	Posterior-most point on the frontal bone at the midline (left)
6	N/A	Posterior-most point on the frontal bone at the midline (right)
7	N/A	Anterior-most point on the parietal bone at the midline (left)
8	N/A	Anterior-most point on the parietal bone at the midline (right)
9	N/A	Maximum curvature point on the posterior part of the parietal bone (left)
10	N/A	Maximum curvature point on the posterior part of the parietal bone (right)
11	N/A	Anterior-most point on the interparietal bone at the midline
12	N/A	Anterior-most point on the occipital bone at the midline

22	N/A	Joining of squamosal body to zygomatic process of squamosal (left)
23	N/A	Tip of the post-tympanic hook (left)
33	N/A	Joining of squamosal body to zygomatic process of squamosal (right)
34	N/A	Tip of the post-tympanic hook (right)

Supplementary Table S5. Anatomical definition of mandible landmarks.

Landmark number	Anatomical definition
1	Tip of the coronoid process (left)
2	Anterior-most point on mandibular condyle (left)
3	Posterior-most point on mandibular condyle (left)
4	Mandible angle (left)
5	Superior-most point on inferior border of mandibular ramus (left)
6	Inferior-most point on border of ramus inferior to incisor alveolar (left)
7	Inferior-most point on incisor alveolar rim (left)
8	Superior-most point on incisor alveolar rim (left)
9	Mandibular foramen (left)
10	Anterior point on molar alveolar rim (left)
11	Intersection of molar alveolar rim and base of coronoid process (left)
12	Tip of the coronoid process (right)
13	Anterior-most point on mandibular condyle (right)
14	Posterior-most point on mandibular condyle (right)
15	Mandible angle (right)
16	Superior-most point on inferior border of mandibular ramus (right)
17	Inferior-most point on border of ramus inferior to incisor alveolar (right)
18	Inferior-most point on incisor alveolar rim (right)
19	Superior-most point on incisor alveolar rim (right)

20	Mandibular foramen (right)
21	Anterior point on molar alveolar rim (right)
22	Intersection of molar alveolar rim and base of coronoid process (right)

Supplementary Table S6. Anatomical definition of humerus landmarks. N/A indicates that the landmarks were not acquired at this stage.

Landmark number PD3	Landmark number PD14 and PD29	Anatomical definition
1	N/A	Intersection between the surface of the head and the medial concavity
2	N/A	Average between the medial-most point of the medial epicondyle and the lateral-most point on the lateral epicondyle
3	3	Medial-most point on the medial epicondyle
4	N/A	Lateral-most point on the lateral epicondyle
N/A	1	Proximal-most point in the humerus head
N/A	2	Deepest point in the radial fossa
N/A	4	Lateral-most point on the epicondyle crest

Supplementary Table S7. Anatomical definition of scapula landmarks.

Landmark number	Anatomical definition
1	Medial-most point on the superior side of the axillary border. Intersection with the glenoid fossa
2	Caudal angle. Intersection between the axillary border and vertebral border.
3	External-most point of the cranial border.

Supplementary Table S8. Overview of normality and homoscedasticity per parameter and test used to evaluate pairwise significance. For both the Shapiro-Wilk and F-test, if one pairwise comparison did not show normality or homoscedasticity the entire parameter was considered not to be and was marked with “No”. M-W = Mann-Whitney test, K-S = Two-Sample Kolmogorov-Smirnov test.

Parameter	Normal according to Shapiro-Wilk?	Equal SD according to F-test?	Test performed
Humerus length high dose PD3	No	Yes	M-W
Humerus length high dose PD14	No	No	K-S
Humerus length high dose PD29	Yes	Yes	Welch's T-test
Humerus width high dose PD3	Yes	No	Welch's T-test
Humerus width high dose PD14	Yes	No	Welch's T-test
Humerus width high dose PD29	Yes	Yes	Welch's T-test
Humerus length low dose PD3	No	Yes	M-W
Humerus length low dose PD14	No	No	K-S
Humerus length low dose PD29	Yes	No	Welch's T-test
Humerus width low dose PD3	No	Yes	M-W
Humerus width low dose PD14	Yes	Yes	Welch's T-test
Humerus width low dose PD29	Yes	Yes	Welch's T-test
Scapula length high dose PD3	Yes	Yes	Welch's T-test
Scapula length high dose PD14	No	No	K-S
Scapula length high dose PD29	Yes	Yes	Welch's T-test
Scapula width high dose PD3	Yes	Yes	Welch's T-test
Scapula width high dose PD14	No	Yes	M-W
Scapula width high dose PD29	Yes	Yes	Welch's T-test
Scapula length low dose PD3	Yes	Yes	Welch's T-test
Scapula length low dose PD14	Yes	Yes	Welch's T-test
Scapula length low dose PD29	No	Yes	M-W
Scapula width low dose PD3	Yes	Yes	Welch's T-test
Scapula width low dose PD14	Yes	Yes	Welch's T-test
Scapula width low dose PD29	No	Yes	M-W

Humerus length high vs low dose PD3	Yes	No	Welch's T-test
Humerus length high vs low dose PD14	Yes	No	Welch's T-test
Humerus length high vs low dose PD29	Yes	Yes	Welch's T-test
Humerus width high vs low dose PD3	No	No	K-S
Humerus width high vs low dose PD14	Yes	Yes	Welch's T-test
Humerus width high vs low dose PD29	Yes	Yes	Welch's T-test
Scapula length high vs low dose PD3	Yes	Yes	Welch's T-test
Scapula length high vs low dose PD14	No	Yes	M-W
Scapula length high vs low dose PD29	No	Yes	M-W
Scapula width high vs low dose PD3	Yes	Yes	Welch's T-test
Scapula width high vs low dose PD14	No	Yes	M-W
Scapula width high vs low dose PD29	No	Yes	M-W
BMD humerus high dose PD3	No	No	K-S
BMD humerus high dose PD14	Yes	Yes	Welch's T-test
BMD humerus high dose PD29	Yes	Yes	Welch's T-test
BMD premaxilla high dose PD3	Yes	No	Welch's T-test
BMD premaxilla high dose PD14	Yes	Yes	Welch's T-test
BMD premaxilla high dose PD29	Yes	No	Welch's T-test
BMD humerus low dose PD3	No	Yes	M-W
BMD humerus low dose PD14	No	Yes	M-W
BMD humerus low dose PD29	Yes	Yes	Welch's T-test
BMD premaxilla low dose PD3	No	No	K-S
BMD premaxilla low dose PD14	No	No	K-S
BMD premaxilla low dose PD29	No	Yes	M-W
BMD humerus high vs low dose PD3	Yes	No	Welch's T-test
BMD humerus high vs low dose PD14	No	Yes	M-W
BMD humerus high vs low dose PD29	Yes	Yes	Welch's T-test
BMD premaxilla high vs low dose PD3	No	Yes	M-W

BMD premaxilla high vs low dose PD14	No	Yes	M-W
BMD premaxilla high vs low dose PD29	No	No	K-S

Supplementary Table S9. *P*s resulting from the permutation tests (10,000 permutation rounds) for the Procrustes distances among groups for the facial shape at the high dose treatment. Bold font indicates statistically significant values.

PD3	TS Treated high dose	TS Untreated	WT Treated high dose
TS Untreated	0.1990		
WT Treated high dose	0.1619	0.0540	
WT Untreated	0.0999	0.1908	0.1650
PD14	TS Treated high dose	TS Untreated	WT Treated high dose
TS Untreated	0.5823		
WT Treated high dose	0.2887	**0.0354**	
WT Untreated	**0.0141**	**0.0365**	**0.0005**
PD29	TS Treated high dose	TS Untreated	WT Treated high dose
TS Untreated	0.0692		
WT Treated high dose	0.2892	0.0540	
WT Untreated	**0.0014**	**0.0141**	**0.0199**

Supplementary Table S10. *P*s resulting from the permutation tests (10,000 permutation rounds) for the Procrustes distances among groups for the facial shape at the low dose treatment. Bold font indicates statistically significant values.

PD3	TS Treated low dose	TS Untreated	WT Treated low dose
TS Untreated	0.0535		
WT Treated low dose	**0.0258**	**0.0016**	
WT Untreated	**0.0187**	0.5516	**0.0010**
PD14	TS Treated low dose	TS Untreated	WT Treated low dose
TS Untreated	**0.0116**		
WT Treated low dose	**0.0004**	**<0.0001**	

WT Untreated	**0.0008**	0.1094	**<0.0001**
PD29	TS Treated low dose	TS Untreated	WT Treated low dose
TS Untreated	0.3125		
WT Treated low dose	**0.0012**	**0.0001**	
WT Untreated	0.4596	**0.0427**	**<0.0001**

Supplementary Table S11. *P*s resulting from the permutation tests (10,000 permutation rounds) for the Procrustes distances among groups for the skull shape at the high dose treatment. Bold font indicates statistically significant values.

PD3	TS Treated high dose	TS Untreated	WT Treated high dose
TS Untreated	0.3120		
WT Treated high dose	**0.0003**	**0.0437**	
WT Untreated	**<0.0001**	**0.0042**	0.0519
PD14	TS Treated high dose	TS Untreated	WT Treated high dose
TS Untreated	0.2963		
WT Treated high dose	**0.0019**	**0.0003**	
WT Untreated	**<0.0001**	**<0.0001**	**<0.0001**
PD29	TS Treated high dose	TS Untreated	WT Treated high dose
TS Untreated	0.1501		
WT Treated high dose	**0.0026**	**0.0027**	
WT Untreated	**<0.0001**	**0.0002**	**0.0281**

Supplementary Table S12. *P*s resulting from the permutation tests (10,000 permutation rounds) for the Procrustes distances among groups for the skull shape at the low dose treatment. Bold font indicates statistically significant values.

PD3	TS Treated low dose	TS Untreated	WT Treated low dose
TS Untreated	0.1427		
WT Treated low dose	**<0.0001**	**<0.0001**	
WT Untreated	**<0.0001**	**<0.0001**	**0.0022**

PD14	TS Treated low dose	TS Untreated	WT Treated low dose
TS Untreated	**0.0472**		
WT Treated low dose	**<0.0001**	**<0.0001**	
WT Untreated	**<0.0001**	**0.0003**	**<0.0001**
PD29	TS Treated low dose	TS Untreated	WT Treated low dose
TS Untreated	**0.0011**		
WT Treated low dose	**<0.0001**	**<0.0001**	
WT Untreated	**<0.0001**	**0.0006**	**<0.0001**

Supplementary Table S13. ***P*****s resulting from the permutation tests (10,000 permutation rounds) for the Procrustes distances among groups for the mandibular shape at the high dose treatment.** Bold font indicates statistically significant values.

PD3	TS Treated high dose	TS Untreated	WT Treated high dose
TS Untreated	0.0563		
WT Treated high dose	**<0.0001**	**<0.0001**	
WT Untreated	**<0.0001**	**0.0002**	**0.0204**
PD14	TS Treated high dose	TS Untreated	WT Treated high dose
TS Untreated	**0.0007**		
WT Treated high dose	**0.0020**	**0.0180**	
WT Untreated	**<0.0001**	**<0.0001**	**<0.0001**
PD29	TS Treated high dose	TS Untreated	WT Treated high dose
TS Untreated	0.2529		
WT Treated high dose	**0.0008**	**0.0104**	
WT Untreated	**<0.0001**	**0.0001**	0.0751

Supplementary Table S14. ***P*****s resulting from the permutation tests (10,000 permutation rounds) for the Procrustes distances among groups for the mandibular shape at the low dose treatment.** Bold font indicates statistically significant values.

PD3	TS Treated low dose	TS Untreated	WT Treated low dose
TS Untreated	**0.0169**		
WT Treated low dose	**<0.0001**	**<0.0001**	
WT Untreated	**<0.0001**	**0.0002**	**<0.0001**
PD14	TS Treated low dose	TS Untreated	WT Treated low dose
TS Untreated	**0.0023**		
WT Treated low dose	**<0.0001**	**<0.0001**	
WT Untreated	**<0.0001**	**<0.0001**	**<0.0001**
PD29	TS Treated low dose	TS Untreated	WT Treated low dose
TS Untreated	**0.0002**		
WT Treated low dose	**<0.0001**	**<0.0001**	
WT Untreated	**0.0001**	**<0.0001**	**<0.0001**

Supplementary Table S15. ***P*****s resulting from the pairwise tests for the humerus length at the high dose treatment.** Bold font indicates statistically significant values according to the Benjamini–Hochberg correction.

PD3	TS Treated high dose	TS Untreated	WT Treated high dose
TS Untreated	0.7210		
WT Treated high dose	0.5878		
WT Untreated	0.1349	0.0401	0.5045
PD14	TS Treated high dose	TS Untreated	WT Treated high dose
TS Untreated	0.0870		
WT Treated high dose	0.0828		
WT Untreated	**0.0027**	**0.0025**	**0.0017**
PD29	TS Treated high dose	TS Untreated	WT Treated high dose
TS Untreated	0.0783		

WT Treated high dose	**0.0044**		
WT Untreated	**<0.0001**	**0.0041**	**0.0013**

Supplementary Table S16. *P*s resulting from the pairwise tests for the humerus length at the low dose treatment. Bold font indicates statistically significant values according to the Benjamini–Hochberg correction.

PD3	TS Treated low dose	TS Untreated	WT Treated low dose
TS Untreated	0.1079		
WT Treated low dose	**0.0003**		
WT Untreated	**0.0024**	0.0401	0.5393
PD14	TS Treated low dose	TS Untreated	WT Treated low dose
TS Untreated	**0.0226**		
WT Treated low dose	**0.0035**		
WT Untreated	**0.0002**	**0.0025**	**0.0033**
PD29	TS Treated low dose	TS Untreated	WT Treated low dose
TS Untreated	0.1161		
WT Treated low dose	**0.0216**		
WT Untreated	**0.0072**	**0.0041**	**0.0044**

Supplementary Table S17. *P*s resulting from the pairwise tests for the humerus width at the high dose treatment. Bold font indicates statistically significant values according to the Benjamini–Hochberg correction.

PD3	TS Treated high dose	TS Untreated	WT Treated high dose
TS Untreated	0.6864		
WT Treated high dose	0.5572		
WT Untreated	0.1559	**0.0178**	0.3720
PD14	TS Treated high dose	TS Untreated	WT Treated high dose
TS Untreated	0.5096		
WT Treated high dose	0.1500		

WT Untreated	**0.0025**	**<0.0001**	**0.0109**
PD29	TS Treated high dose	TS Untreated	WT Treated high dose
TS Untreated	**0.0288**		
WT Treated high dose	**0.0002**		
WT Untreated	**<0.0001**	**<0.0001**	**0.0013**

Supplementary Table S18. *P*s resulting from the pairwise tests for the humerus width at the low dose treatment. Bold font indicates statistically significant values according to the Benjamini–Hochberg correction.

PD3	TS Treated low dose	TS Untreated	WT Treated low dose
TS Untreated	0.0813		
WT Treated low dose	**<0.0001**		
WT Untreated	**0.0003**	**0.0105**	0.7748
PD14	TS Treated low dose	TS Untreated	WT Treated low dose
TS Untreated	0.0697		
WT Treated low dose	**<0.0001**		
WT Untreated	**<0.0001**	**<0.0001**	**0.0144**
PD29	TS Treated low dose	TS Untreated	WT Treated low dose
TS Untreated	**0.0291**		
WT Treated low dose	**0.0001**		
WT Untreated	**<0.0001**	**<0.0001**	**0.0310**

Supplementary Table S19. *P*s resulting from the pairwise tests for the scapula length at the high dose treatment. Bold font indicates statistically significant values according to the Benjamini–Hochberg correction.

PD3	TS Treated high dose	TS Untreated	WT Treated high dose
TS Untreated	0.6053		
WT Treated high dose	0.4756		
WT Untreated	0.1529	0.2079	0.3019

PD14	TS Treated high dose	TS Untreated	WT Treated high dose
TS Untreated	0.1181		
WT Treated high dose	0.3267		
WT Untreated	**0.0108**	**0.0018**	**0.0002**
PD29	TS Treated high dose	TS Untreated	WT Treated high dose
TS Untreated	0.0304		
WT Treated high dose	**0.0084**		
WT Untreated	**<0.0001**	**0.0024**	**<0.0001**

Supplementary Table S20. *P*s resulting from the pairwise tests for the scapula length at the low dose treatment. Bold font indicates statistically significant values according to the Benjamini–Hochberg correction.

PD3	TS Treated low dose	TS Untreated	WT Treated low dose
TS Untreated	**0.0224**		
WT Treated low dose	**0.0003**		
WT Untreated	**0.0006**	0.2079	0.4423
PD14	TS Treated low dose	TS Untreated	WT Treated low dose
TS Untreated	**0.0202**		
WT Treated low dose	**0.0034**		
WT Untreated	**0.0008**	**0.0012**	**0.0121**
PD29	TS Treated low dose	TS Untreated	WT Treated low dose
TS Untreated	0.0519		
WT Treated low dose	**0.0001**		
WT Untreated	**<0.0001**	**<0.0001**	0.3262

Supplementary Table S21. ***P*s resulting from the pairwise tests for the scapula width at the high dose treatment.** Bold font indicates statistically significant values according to the Benjamini–Hochberg correction.

PD3	TS Treated high dose	TS Untreated	WT Treated high dose
TS Untreated	0.7030		
WT Treated high dose	0.0648		
WT Untreated	0.0455	0.0799	0.9657
PD14	TS Treated high dose	TS Untreated	WT Treated high dose
TS Untreated	0.1520		
WT Treated high dose	0.0853		
WT Untreated	**0.0003**	**0.0112**	**0.0082**
PD29	TS Treated high dose	TS Untreated	WT Treated high dose
TS Untreated	**0.0243**		
WT Treated high dose	**<0.0001**		
WT Untreated	**<0.0001**	**0.0049**	**0.0006**

Supplementary Table S22. ***P*s resulting from the pairwise tests for the scapula width at the low dose treatment.** Bold font indicates statistically significant values according to the Benjamini–Hochberg correction.

PD3	TS Treated low dose	TS Untreated	WT Treated low dose
TS Untreated	0.0539		
WT Treated low dose	**<0.0001**		
WT Untreated	**<0.0001**	0.0799	**0.0277**
PD14	TS Treated low dose	TS Untreated	WT Treated low dose
TS Untreated	**0.0167**		
WT Treated low dose	**0.0035**		
WT Untreated	**0.0007**	**0.0114**	**0.0343**
PD29	TS Treated low dose	TS Untreated	WT Treated low dose
TS Untreated	0.0519		

WT Treated low dose	**0.0001**		
WT Untreated	**<0.0001**	**0.0002**	**0.0292**

Supplementary Table S23. *P*s resulting from the pairwise tests for the bone mineral density of the humerus at the high dose treatment. Bold font indicates statistically significant values according to the Benjamini–Hochberg correction.

PD3	TS Treated high dose	TS Untreated	WT Treated high dose
TS Untreated	0.7301		
WT Treated high dose	0.4182		
WT Untreated	0.0493	**0.0097**	0.1641
PD14	TS Treated high dose	TS Untreated	WT Treated high dose
TS Untreated	0.8467		
WT Treated high dose	**0.0074**		
WT Untreated	**0.0157**	**0.0003**	0.3861
PD29	TS Treated high dose	TS Untreated	WT Treated high dose
TS Untreated	0.4238		
WT Treated high dose	0.1202		
WT Untreated	**0.0024**	0.1827	0.0755

Supplementary Table S24. *P*s resulting from the pairwise tests for the bone mineral density of the humerus at the low dose treatment. Bold font indicates statistically significant values according to the Benjamini–Hochberg correction.

PD3	TS Treated low dose	TS Untreated	WT Treated low dose
TS Untreated	>0.9999		
WT Treated low dose	0.2300		
WT Untreated	**0.0117**	**0.0062**	0.1083
PD14	TS Treated low dose	TS Untreated	WT Treated low dose
TS Untreated	**0.0295**		
WT Treated low dose	**0.0037**		

WT Untreated	**<0.0001**	**0.0001**	**0.0035**
PD29	TS Treated low dose	TS Untreated	WT Treated low dose
TS Untreated	0.3557		
WT Treated low dose	**0.0018**		
WT Untreated	**0.0009**	0.1827	0.4503

Supplementary Table S25. *P*s resulting from the pairwise tests for the bone mineral density of the premaxilla at the high dose treatment. Bold font indicates statistically significant values according to the Benjamini–Hochberg correction.

PD3	TS Treated high dose	TS Untreated	WT Treated high dose
TS Untreated	0.3968		
WT Treated high dose	**0.0010**		
WT Untreated	**0.0032**	**0.0025**	0.6706
PD14	TS Treated high dose	TS Untreated	WT Treated high dose
TS Untreated	0.7624		
WT Treated high dose	0.1205		
WT Untreated	**0.0016**	**0.0031**	0.2853
PD29	TS Treated high dose	TS Untreated	WT Treated high dose
TS Untreated	**0.0067**		
WT Treated high dose	0.1304		
WT Untreated	**<0.0001**	0.0331	**0.0001**

Supplementary Table S26. *P*s resulting from the pairwise tests for the bone mineral density of the premaxilla at the low dose treatment. Bold font indicates statistically significant values according to the Benjamini–Hochberg correction.

PD3	TS Treated low dose	TS Untreated	WT Treated low dose
TS Untreated	0.3017		
WT Treated low dose	**0.0021**		
WT Untreated	**0.0031**	**0.0295**	0.1207

PD14	TS Treated low dose	TS Untreated	WT Treated low dose
TS Untreated	**0.0226**		
WT Treated low dose	**0.0102**		
WT Untreated	**0.0002**	**0.0062**	**0.0055**
PD29	TS Treated low dose	TS Untreated	WT Treated low dose
TS Untreated	0.0635		
WT Treated low dose	**0.0077**		
WT Untreated	**0.0002**	**0.0118**	**0.0001**
